# Distribution of Intravenously Administered Acetylcholinesterase Inhibitor and Acetylcholinesterase Activity in the Adrenal Gland: ^11^C-Donepezil PET Study in the Normal Rat

**DOI:** 10.1371/journal.pone.0107427

**Published:** 2014-09-16

**Authors:** Tadashi Watabe, Sadahiro Naka, Hayato Ikeda, Genki Horitsugi, Yasukazu Kanai, Kayako Isohashi, Mana Ishibashi, Hiroki Kato, Eku Shimosegawa, Hiroshi Watabe, Jun Hatazawa

**Affiliations:** 1 Department of Molecular Imaging in Medicine, Osaka University Graduate School of Medicine, Suita, Osaka, Japan; 2 PET molecular Imaging Center, Osaka University Graduate School of Medicine, Suita, Osaka, Japan; 3 Osaka University Hospital, Suita, Osaka, Japan; 4 Department of Nuclear Medicine and Tracer Kinetics, Osaka University Graduate School of Medicine, Suita, Osaka, Japan; 5 Immunology Frontier Research Center, Osaka University, Suita, Osaka, Japan; Weizmann Institute of Science, Israel

## Abstract

**Purpose:**

Acetylcholinesterase (AChE) inhibitors have been used for patients with Alzheimer's disease. However, its pharmacokinetics in non-target organs other than the brain has not been clarified yet. The purpose of this study was to evaluate the relationship between the whole-body distribution of intravenously administered ^11^C-Donepezil (DNP) and the AChE activity in the normal rat, with special focus on the adrenal glands.

**Methods:**

The distribution of ^11^C-DNP was investigated by PET/CT in 6 normal male Wistar rats (8 weeks old, body weight  = 220±8.9 g). A 30-min dynamic scan was started simultaneously with an intravenous bolus injection of ^11^C-DNP (45.0±10.7 MBq). The whole-body distribution of the ^11^C-DNP PET was evaluated based on the Vt (total distribution volume) by Logan-plot analysis. A fluorometric assay was performed to quantify the AChE activity in homogenized tissue solutions of the major organs.

**Results:**

The PET analysis using Vt showed that the adrenal glands had the 2nd highest level of ^11^C-DNP in the body (following the liver) (13.33±1.08 and 19.43±1.29 ml/cm^3^, respectively), indicating that the distribution of ^11^C-DNP was the highest in the adrenal glands, except for that in the excretory organs. The AChE activity was the third highest in the adrenal glands (following the small intestine and the stomach) (24.9±1.6, 83.1±3.0, and 38.5±8.1 mU/mg, respectively), indicating high activity of AChE in the adrenal glands.

**Conclusions:**

We demonstrated the whole-body distribution of ^11^C-DNP by PET and the AChE activity in the major organs by fluorometric assay in the normal rat. High accumulation of ^11^C-DNP was observed in the adrenal glands, which suggested the risk of enhanced cholinergic synaptic transmission by the use of AChE inhibitors.

## Introduction

Acetylcholinesterase (AChE) is one of the most crucial enzymes in the nervous system. AChE is a tetrameric serine hydrolase that rapidly degrades the neurotransmitter acetylcholine (ACh) into choline and acetate [1]. It is mainly found at the neuromuscular junctions and cholinergic synapses in the central nervous system, where its activity serves to terminate synaptic transmission. Progressive loss of cholinergic neurons is observed in Alzheimer's disease (AD) patients with severe memory loss and impairment of cognitive function [2], and AChE inhibitors have been used for such patients to protect against the reduction of acetylcholine in the synapses and to enhance cholinergic activity in the affected regions of the brain [3]. Recently, various types of AChE inhibitors have been used for the treatment of AD, such as donepezil (DNP), galantamine, and rivastigmine [4]. Among these, DNP is the most well-known, with a 15-year history of clinical use in AD patients.

Micro-dose positron emission tomography (PET), in which radiolabeled drugs are administered in amounts far below the pharmacological dose, enables direct investigation of the pharmacokinetics of drugs [5]. Micro-dose PET is useful for drug development, especially for prediction of the adverse effects by unexpected distribution of the drug to non-target organs. A previous study reported on the pharmacokinetics of AChE inhibitors in the brain using a radiolabeled tracer. Funaki et al. reported that the regional distribution of ^11^C-DNP in the rat brain was heterogeneous and that the binding of ^11^C-DNP was higher in the brain stem and striatum and lower in the cerebellum by autoradiographic method [6]. Okamura et al. demonstrated that intravenously administered ^11^C-donepezil rapidly entered the brain, becoming distributed mainly in the striatum, thalamus and cerebellum, which are known to contain AChE at high concentrations as compared to the cerebral cortex and hippocampus [7]. The uptake of ^11^C-DNP in the brain was fully evaluated by previous studies not only in rats as well as in humans. However, the pharmacokinetics of AChE inhibitors in non-target organs other than the brain has not been clarified yet. The purpose of this study was to evaluate the relationship between the whole-body distribution of intravenously administered ^11^C- DNP by PET and the activity of AChE in specific organs by fluorometric assay, with special focus on the adrenal glands, in the normal rat.

## Materials and Methods

### Preparation of ^11^C-DNP

^11^C-DNP was synthesized as described previously [6]. Briefly, the 5′-O-desmethylprecursor was dissolved in acetone, followed by the addition of 0.1 M NaOH. ^11^C-Methyl iodide was prepared from ^11^C-CO_2_ and converted to ^11^C-methyl triflate (^11^C-MeOTf). ^11^C-Donepezil was produced in a reaction vessel from ^11^C-MeOTf and purified by high-performance liquid chromatography (HPLC). The radioactivity obtained was 1.86–2.54 GBq, with a specific activity of 56–120 GBq/µmol at the end of the synthesis (30–35 min from the end of the ^11^C production). The radiochemical purity was greater than 98%.

### Animal preparation and PET measurements

Normal male Wistar rats from Japan SLC Inc. (Hamamatsu, Japan) were used for this study. The animals were housed under a 12-h light/12-h dark cycle and had free access to food and water. Six rats (8 weeks old, body weight  = 220±8.9 g) were anesthetized with 2% isoflurane plus 100% oxygen and a Terumo 24 G indwelling cannula was inserted into the tail vein. PET data were acquired by means of small-animal PET (Inveon PET/CT system, Siemens Medical Solutions, Knoxville, USA) [8]. The animals were placed in a feet-first prone position in the PET scanner. ^11^C-DNP (45.0±10.7 MBq) was administered intravenously via the catheter cannula, and dynamic PET measurements (list-mode acquisition) were started at the same time in two bed positions (axial FOV = 127 mm). The scan area and acquisition time per bed position were as follows; head to abdomen for the first 30 min and chest to hind limbs for the next 10 min (n = 3), chest to hind limbs for the first 30 min and head to upper abdomen for the next 10 min (n = 3). The body temperature was maintained by the use of a heating sheet system on the bed. CT of the whole body was performed for the attenuation and scatter correction. The PET data were reconstructed by the 2-dimensional ordered subset expectation maximization method (2D-OSEM). The image matrix was 128×128×159, which yielded a voxel size of 0.776×0.776×0.796 mm. All animal experiments were performed in compliance with the guidelines of the Institute of Experimental Animal Sciences of the Osaka University Graduate School of Medicine. The protocol was approved by the Animal Care and Use Committee of the Osaka University Graduate School of Medicine (Approval number: 20-144-008) and all efforts were made to minimize suffering. Euthanasia was performed by deep inhalation anesthesia after the PET/CT study.

### Data analysis

The resulting sinograms were reconstructed into 30 frames (30 frame ×60 sec) for generation of the time-activity curves. Regions of interest were manually placed in the cerebrum, cerebellum, submandibular glands, lungs, thymus, heart, liver, stomach, small intestine, spleen, kidneys, adrenal glands and the blood pool inside the left ventricle on the summed PET-CT images. The total distribution volume (Vt) was calculated by Logan plot analysis using the PMOD software (version 3.0; PMOD Technologies Ltd.). The image input from the blood pool inside the left ventricle was used as a substitute for the plasma input. Regional uptake of radioactivity was decay-corrected to the injection time and expressed as the standardized uptake value (SUV), corrected for the injected dose (MBq) and body weight (g).

### Measurement by well counter and metabolite analysis

The animals were sacrificed and dissection was performed immediately after the PET/CT acquisition (n = 4). The following organs were collected: the cerebrum, cerebellum, submandibular glands, lung, thymus, heart, liver, stomach, small intestine, pancreas, spleen, kidneys, adrenal glands, and blood. The weight of each of the organs and the radioactivity levels were measured with a well scintillation counter (BeWell, Molecular Imaging Labo, Osaka, Japan), and the radioactivity (cps: count per second) was corrected for the decay from the injection time. The radioactivity concentrations were divided by the sample weight and injected dose for comparison (cps/g/MBq). Metabolite analysis in the blood samples was performed using HPLC and a gamma counter for fraction measurements (ARC 7001, Hitachi Aloka Medical, Ltd).

### Fluorometric assay

The AChE activities and ACh levels were quantified using the AmpliteTM Fluorimetric Acetylcholinesterase Assay Kit (AAT Bioquest, Inc). In this assay, AChE activity was quantified by fluorescence measurement which detected the choline produced from the hydrolysis of ACh by AChE through choline oxidase-mediated enzyme coupling reactions. Small samples from each organ were collected from the dissected animals (n = 3) and the sample weights were measured. PBS solution was added at a volume of 1000 µl to each of the samples placed in tubes and homogenization was performed under ice-cooling using the homogenizer (Viola-homogenizer VH-10, VIOLAMO). The supernatants were collected as the tissue extracts after the centrifugation (5000 rotations per minute, 10 min). Serially diluted AChE and ACh standard solutions (from 0.1 to 100 mU/mL) and tissue extracts were pipetted into 96-well microplates, followed by the addition of an acetylthiocholine reaction mixture. After 20 to 30 min of incubation, the fluorescence intensities were measured with a fluorescence microplate reader (SH-9000, Corona). The AChE activities (mU/mg) and ACh levels (mU/mg) were calculated by the plotting from each standard curve.

### Statistics

Correlations between the two parameters were determined by by Pearson's rank test for the parametric data and Spearman's rank test for the non-parametric data, followed by the Shapiro-Wilk test for normal distribution. Probability values of less than 0.05 were considered to denote statistical significance.

## Results

**Figure 1:**
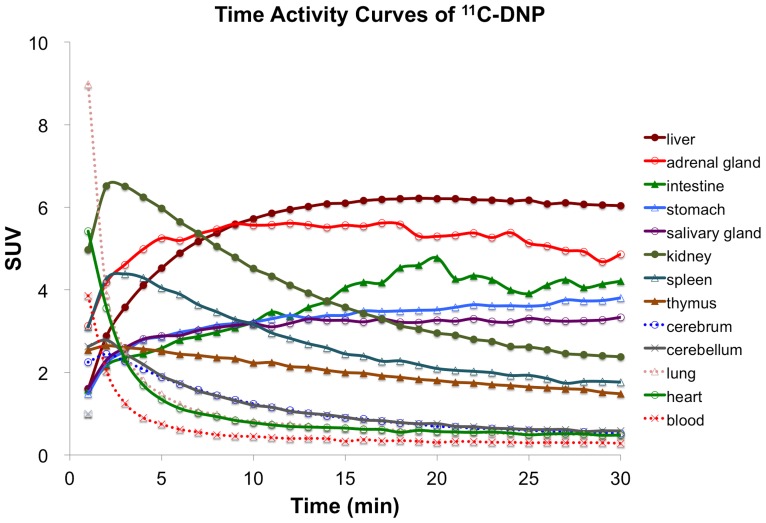
Time activity curves of major organs on ^11^C-DNP PET.

**Figure 2:**
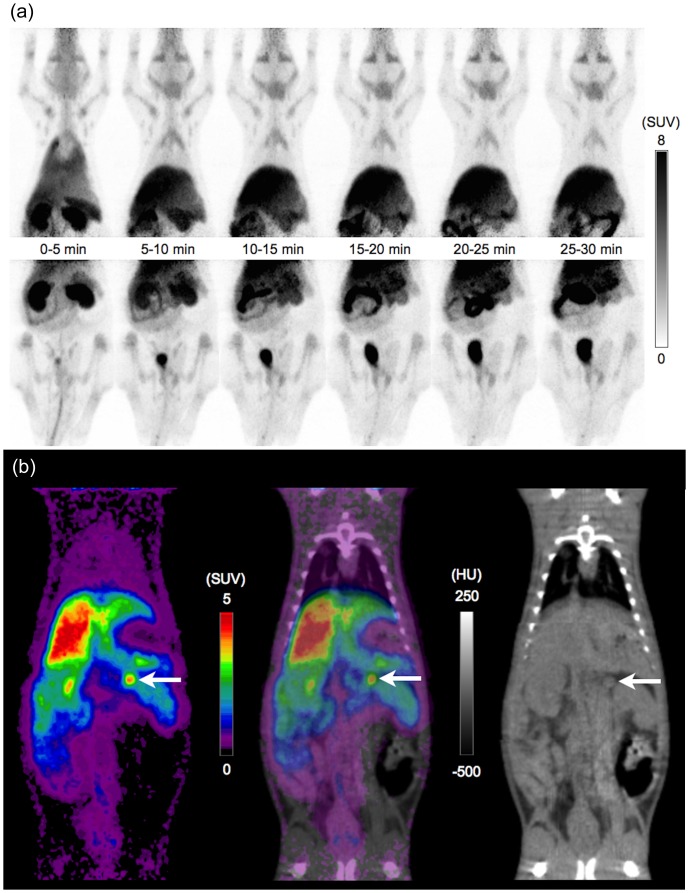
Whole body images after the administration of ^11^C-DNP: (a) dynamic maximum intensity projection images of PET, (b) coronal images of PET, CT, and PET/CT (20–40 min, arrow: left adrenal gland).

The time-activity curves in the major organs are shown in Fig. 1. The following pharmacokinetic characteristics were observed; washout pattern in the lungs and heart, rapid increase and washout pattern in the cerebrum, cerebellum, kidneys and spleen, rapid increase and a slow decrease pattern in the thymus, gradual increase up to 20 min and steady pattern in the liver and submandibular glands, gradual increase in the stomach, and gradual increase and variable high accumulation in the small intestine, suggesting enterohepatic circulation. The adrenal glands showed relatively high accumulation (peak SUV mean  = 5.6) and a gradual increase and slow decrease pattern. The whole-body ^11^C-DNP PET images are shown in Fig. 2.

The determined values of Vt by Logan plot analysis of the PET images, SUVs at 30 min, radioactivity counts determined by the well counter, and AChE activities determined by fluorometric assay are shown in Table 1. Analysis of the PET images showed that the Vt was the highest in the liver, followed by that in the adrenal glands, stomach, salivary glands, and small intestine. The SUVs at 30 min showed the same trend as the Vt. Well counts after the PET study showed the highest accumulation in the adrenal glands, followed by the pancreas. The AChE activity was the highest in the small intestine, followed by that in the stomach and adrenal glands.

**Table 1:** The relationships among Vt by Logan plot analysis, SUV at 30 min, radioactivity count by well counter, and AChE activity by fluorometric assay.

	Vt	SUV	Well count	AChE activity
	(ml/cm^3^)	at 30 min	(kcps/g/MBq)	(mU/mg)
Lung	2.02±0.28	0.49±0.11	1.66±0.19	11.8±4.9
Thymus	4.42±0.47	1.48±0.29	1.76±0.18	16.2±3.6
Heart	1.81±0.26	0.48±0.11	0.47±0.06	13.5±6.5
Liver	19.43±1.29	6.04±1.58	6.42±1.11	13.5±4.2
Stomach	11.07±0.89	3.80±1.29	2.49±1.59	38.5±8.1
Small intestine	8.35±1.73	4.21±1.24	5.28±4.86	83.1±3.0
Pancreas	(N.A.)	(N.A.)	8.35±1.99	14.4±2.0
Spleen	5.23±0.20	1.76±0.43	1.82±0.20	18.9±1.7
Kidney	7.83±0.98	2.38±0.63	2.27±0.37	14.4±1.4
Adrenal gland	13.33±1.08	4.86±1.30	9.36±2.44	24.9±1.6
Salivary gland	8.64±0.96	3.33±0.37	3.69±0.46	10.1±2.2
Cerebrum	1.75±0.10	0.52±0.02	0.35±0.05	16.1±3.6
Cerebellum	1.82±0.11	0.58±0.04	0.35±0.03	18.2±5.6

**Figure 3:**
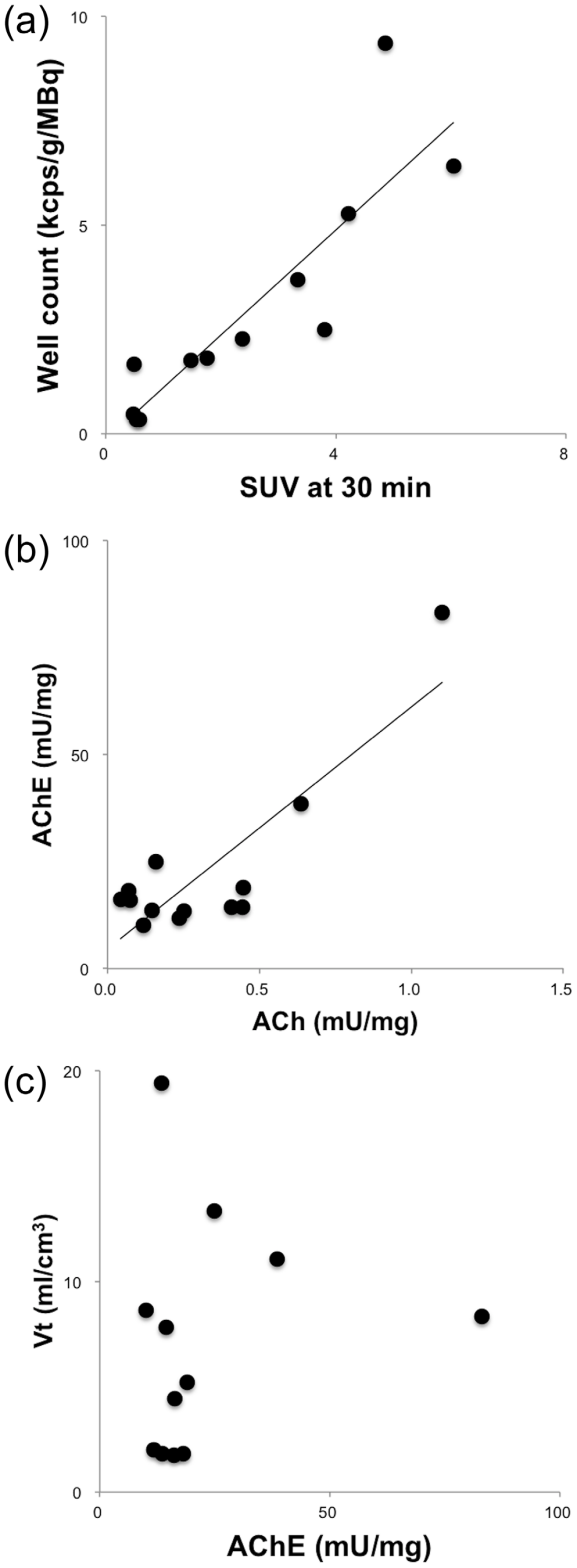
Relationships between (a) well count and SUV (R = 0.93, p<0.001), (b) AChE activity and ACh level (R = 0.86, p<0.001), and (c) Vt and AChE activity (R = 0.17, p = 0.60).

The relationships among the Vt values, well counts, AChE activities and ACh levels are shown in Fig. 3. Significant correlations were observed between the well counts and SUVs at 30 min, AChE activities and ACh levels, whereas no significant relationship was observed between the Vt values and AChE activities.

Metabolite analysis showed that 24.1±0.9% of the ^11^C-DNP in the plasma remained unchanged at approximately 1 hour after the administration.

## Discussion

We investigated the distribution of the AChE inhibitor drug DNP by the micro-dose PET technique. The dynamic PET images showed that ^11^C-DNP was metabolized in the body and excreted via the liver and the kidney. On the ^11^C-DNP PET images, high accumulation was observed in the excretory organs (liver and kidneys), digestive tract (stomach and small intestine), and glandular organs (adrenal glands, pancreas, and salivary glands). Among these, the highest distribution of ^11^C-DNP was observed in the adrenal glands, except for that in the excretory organs. Dynamic PET images showed the kidney had the highest accumulation in the early phase (0–5 min) after the administration of ^11^C-DNP. This distribution is mainly caused by renal blood flow, and uptake in the kidney was immediately excreted into the bladder. This rapid increase and washout pattern in the kidney was usually observed in many PET studies, and not reflecting the specific binding to AChE. A previous study evaluated the pharmacokinetics and tissue distribution after oral administration of ^3^H-galantamine, an AChE inhibitory drug recently developed for Alzheimer's disease, and reported the following ratios of the 0–24 h area under the curve of tissue to plasma; liver (10.6), kidney (7.47), salivary gland (4.45), adrenal gland (3.32), spleen (2.92), and brain (0.60) [9]. These results were consistent with our results, although the tracer and administration method were different.

Fluorometric assay revealed relatively high AChE activities in the digestive tract and adrenal glands, which might reflect the functions of ACh as the neurotransmitter in the parasympathetic nervous system, such as stimulation of peristalsis in the digestive tract and cholinergic synaptic transmission in the adrenal medulla [10]. A previous study evaluated the AChE activities by Ellman's method in sham-operated Wistar rats, and reported AChE activities of 0.3 nmoles/min/mg in the kidneys, 1.1 in the liver, 0.9 in the pancreas, 3.0 in the stomach, and 4.0 in the spleen [11]. These results were consistent with our results, except for the spleen. We compared the distribution of ^11^C-DNP and AChE activities and found no significant relationship between the Vt values and AChE activities. This difference in the distribution of ^11^C-DNP and AChE activities is considered to be attributable to the influence of metabolites. Matsui et al. reported that the percentage of unchanged DNP at 30 min after oral administration was 90% in the brain, 81% in the liver, 72% in the kidney, and 31% in the plasma [12]. Okamura et al. reported that the regional distribution of ^11^C-donepezil was consistent with the regional AChE activities as determined in a human postmortem study by Finkelstein Y, suggesting selective binding of donepezil to AChE [7], [13]. On the contrary, the regional brain distribution of ^11^C-DNP synthesized from M1 as a precursor did not reflect the measured AChE distribution in the rabbit brain [14]. This may be due to the incorrect labeling position, because demethylation at position 6 can yield a potent unlabeled metabolite (M1), as pointed out by Funaki [6]. These results suggest that a difference in the proportion of metabolites formed might influence the ratio of the ^11^C-DNP distribution to the AChE activity. Funaki also reported that the binding in the hippocampus was not displaced by unlabeled donepezil, indicating the higher non-specific binding of ^11^C-DNP in the hippocampus [6]. Therefore, there is the possibility that accumulation of ^11^C-DNP is higher than the AChE activity in some organs due to high non-specific binding. Recent study reported donepezil bound to sigma-1 receptors in the rat brain, in a dose-dependent manner [15]. There is a possibility that ^11^C-DNP binds not only AChE but also other receptors in the rat brain and body organs, which resulted in the discrepancy between the distribution of ^11^C-DNP and AChE activities.

Kikuchi et al. verified the kinetics in the rat brain of ^18^F-FEP-4MA, showing comparable brain kinetics to that of MP4A, which is an established radio-probe for the measurement of AChE activity *in vivo* by PET [16]. The transfer of the alcoholic metabolite of ^18^F-FEP-4MA, as the alcoholic metabolite of MP4A, from the blood to the brain is limited. These tracers might be better for evaluating the AChE activities in the whole body, although the effects of the alcoholic metabolite of ^18^F-FEP-4MA in other organs are uncertain.

In the micro-dose PET study, the administered dose of the radiolabeled drug is far below the pharmacological dose [5]. We evaluated the whole-body distribution by a planar imaging system, and no significant difference was observed between the use of a micro-dose of the radiolabeled drug and of the pharmacological dose mixed with non-radiolabeled drug (unpublished data). Therefore, micro-dose PET is useful for the prediction of adverse effects of drugs as it allows determination of the distribution in non-target organs. DNP is an AChE inhibitor which prevents degradation of acetylcholine [4]. Excessive inhibition of AChE might influence cholinergic neurotransmission [17]. In the rat adrenal medulla, inhibition of AChE activity not only enhanced cholinergic synaptic transmission, but also elicited muscarinic receptor-mediated synaptic transmission for epinephrine release [11]. AChE inhibitors might exert serious adverse effects by affecting acetylcholine-induced catecholamine release in the adrenal glands. There are a few reports that AChE inhibitors were associated with QT prolongation and torsades de pointes ventricular tachycardia as well as syncope and bradycardia [18]. These adverse effects might be caused by unstable catecholamine release from the adrenal glands. Adverse cardiovascular effects are less common than other adverse gastrointestinal effects, but they should be considered in the susceptible patients.

In the fluorometric assay, the AChE activities in the tissue homogenates were measured by quantifying the amount of thiocholine produced from the hydrolysis of ACh by AChE. Irie et al. evaluated radioactive ACh analogs for mapping brain AChE in vivo [19]. This tracer is metabolized specifically by AChE into hydrophilic metabolite and intra-brain distribution of the tracers reflected a pattern of AChE activity. There is a possibility for the correlation between AChE activity and ACh level in the body organs as well as in the brain. The correlation between AChE vs ACh was highly influenced by a single high point. When the highest one point between AChE vs ACh was removed, the correlation result was R = 0.531 (p = 0.076).

There were some limitations of this study, especially in relation to the analysis in the PET/CT study and of the metabolites. While setting the ROIs on the PET images, it was difficult to identify the pancreas due to the spillover from the high accumulation in the small intestine. Furthermore, the influence of the partial volume effect is not negligible for small organs, such as the adrenal glands. Measurement of the counts by a well counter was performed after the PET study as a substitute or accurate method to evaluate the accumulation in the adrenal glands and the pancreas. Another limitation was that the accumulation in the small intestine was partly affected by the excretion from the biliary tract into the enterohepatic circulation, which resulted in variable accumulation depending on the position of the excreted radioactivity. Yet another limitation was that the proportions of the metabolites in each organ were not evaluated in this study due to the short half-life of ^11^C (20 min). The influence of metabolites should be assessed in future studies to clarify the AChE inhibitory effect in each organ from the distribution of ^11^C-DNP.

In this study, we selected male young rats to prevent the influence of sexual cycle in females and age-related variations according to the previous study which reported the distribution of ^11^C-DNP in the brain [6]. There are reports about a gender difference and influence of aging on drug metabolism [20]. Previous study reported that the brain and plasma concentrations of these compounds are higher in aged than in young rats and the inhibitory effects of donepezil on cholinesterase were more marked in aged than in young rats [21]. It's more appropriate to select the subjects in similar settings of the age and gender as the patients who have been administered the drug. Furthermore, there is a possibility of species difference in pharmacokinetics. The future step of our study is to perform the micro-dose PET in humans.

## Conclusions

This study demonstrated the whole-body distribution of ^11^C-DNP by PET and the AChE activities in specific organs by fluorometric assay in the normal rat. High accumulation of ^11^C-DNP was observed in the adrenal glands, which suggests the risk of adverse effects by enhanced cholinergic synaptic transmission after the administration of AChE inhibitors.

## Supporting Information

Checklist S1 (PDF)
